# Accurate Weed Mapping and Prescription Map Generation Based on Fully Convolutional Networks Using UAV Imagery

**DOI:** 10.3390/s18103299

**Published:** 2018-10-01

**Authors:** Huasheng Huang, Jizhong Deng, Yubin Lan, Aqing Yang, Xiaoling Deng, Sheng Wen, Huihui Zhang, Yali Zhang

**Affiliations:** 1 College of Engineering, South China Agricultural University, Wushan Road, Guangzhou 510642, China; huanghsheng@stu.scau.edu.cn (H.H.); ylzhang@scau.edu.cn (Y.Z.); 2 National Center for International Collaboration Research on Precision Agricultural Aviation Pesticide Spraying Technology, Wushan Road, Guangzhou 510624, China; dengxl@scau.edu.cn (X.D.); vincen@scau.edu.cn (S.W.); 3 College of Electronic Engineering, South China Agricultural University, Wushan Road, Guangzhou 510624, China; yangaqing@stu.scau.edu.cn; 4 Engineering Fundamental Teaching and Training Center, South China Agricultural University, Wushan Road, Guangzhou 510624, China; 5 USDA, Agricultural Research Service, Water Management Research Unit, 2150 Centre Ave., Building D, Suite 320, Fort Collins, CO 80526-8119, USA; huihui.zhang@ars.usda.gov

**Keywords:** UAV, semantic labeling, FCN, weed mapping, prescription map

## Abstract

Chemical control is necessary in order to control weed infestation and to ensure a rice yield. However, excessive use of herbicides has caused serious agronomic and environmental problems. Site specific weed management (SSWM) recommends an appropriate dose of herbicides according to the weed coverage, which may reduce the use of herbicides while enhancing their chemical effects. In the context of SSWM, the weed cover map and prescription map must be generated in order to carry out the accurate spraying. In this paper, high resolution unmanned aerial vehicle (UAV) imagery were captured over a rice field. Different workflows were evaluated to generate the weed cover map for the whole field. Fully convolutional networks (FCN) was applied for a pixel-level classification. Theoretical analysis and practical evaluation were carried out to seek for an architecture improvement and performance boost. A chessboard segmentation process was used to build the grid framework of the prescription map. The experimental results showed that the overall accuracy and mean intersection over union (mean IU) for weed mapping using FCN-4s were 0.9196 and 0.8473, and the total time (including the data collection and data processing) required to generate the weed cover map for the entire field (50 × 60 m) was less than half an hour. Different weed thresholds (0.00–0.25, with an interval of 0.05) were used for the prescription map generation. High accuracies (above 0.94) were observed for all of the threshold values, and the relevant herbicide saving ranged from 58.3% to 70.8%. All of the experimental results demonstrated that the method used in this work has the potential to produce an accurate weed cover map and prescription map in SSWM applications.

## 1. Introduction

Chemical control is necessary to control weed infestation and to ensure rice production [1]. Traditionally, the chemical control strategy has three steps: a single pre-emergence herbicide application, a post-emergence herbicide treatment, and an optional late post-emergence chemical spray [2]. This strategy has been proven to be effective for weed control, through years of rice cultivation applications [3]. However, the consistently increased use of herbicides has caused a negative impact on rice production and the environment [4]. Usually farmers carry out uniform herbicide spraying over the entire field and do not consider the distribution of weed infestations [5]. Excessive herbicides are applied over the areas with an absence of weed infestations, resulting in environmental pollution and chemical residues [1]. Site specific weed management (SSWM) recommends a chemical reduction in the application and utilization of adequate herbicides based on the weed coverage [6]. In the context of SSWM, the prescription map can provide decision making information for a variable-rate spraying machine (i.e., tractors or UAVs), which may reduce the use of herbicides while enhancing their chemical effects [7]. 

However, in order to obtain a prescription map, it is necessary to produce a weed cover map [8]. UAV remote sensing provides a non-destructive and cost-effective platform for rapid monitoring of weed infestations [9]. Compared with other remote sensing platforms (i.e., satellite and piloted aircraft remote sensing), UAV is able to fly at a low altitude and can capture high resolution imagery, which may monitor small weed patches in detail [10]. Several works on weed mapping using UAV remote sensing have been conducted [4,6,8]. Peña et al. [6] used UAV multispectral imagery for weed mapping in maize fields. An automatic object-based image analysis (OBIA) procedure was developed, and a weed cover map was produced with 86% overall accuracy. López-Granados et al. [4] focused on the evaluation of different sensors (red, green, and blue (RGB) and multispectral cameras) and altitudes (30 and 60 m) for weed mapping. A robust image analysis method was developed for weed mapping, and high accuracies were observed using the multispectral camera at any flight altitude. Most of these studies were based on the OBIA framework. The use of the OBIA method requires a process of feature selection, which must be performed by manual designs [6]. Although hand-designed features (i.e., texture features and vegetation indices) [11,12,13] are a proven approach, they are application dependent and hard to generalize [14]. 

Fully convolutional networks (FCN) is an automatic feature learning algorithm that can address the disadvantages of OBIA approaches [15]. FCN implements the forward and backward process in an end-to-end mode, which performs the feature learning automatically [16]. In recent years, FCN has achieved great success in computer vision [16,17] and remote sensing applications [18,19,20]. FCN introduces the pixel-to-pixel translation in an end-to-end mode, which shows great potential for the weed mapping of UAV imagery. However, no related study on weed mapping using FCN can be accessed, except for the work of [21,22]. In these works, semantic labeling approaches were directly applied on the collected UAV imagery. The experimental results showed that the semantic labeling approaches outperformed others in terms of accuracy and efficiency. However, both of these works did not generate a weed cover map or a prescription map for the whole field, which may not satisfy the requirement of practical SSWM applications. The objective of this work is as follows: to (1) compare the performance of different workflows so as to generate a weed cover map for the whole field; (2) carry out the theoretical analysis and practical evaluation to seek for an architecture improvement of FCN, which may bring a performance boost in accuracy and efficiency; and (3) generate a prescription map, which may provide decision-making information for SSWM applications.

## 2. Data Collection

### 2.1. Study Field and Data Collection

**Figure 1:**
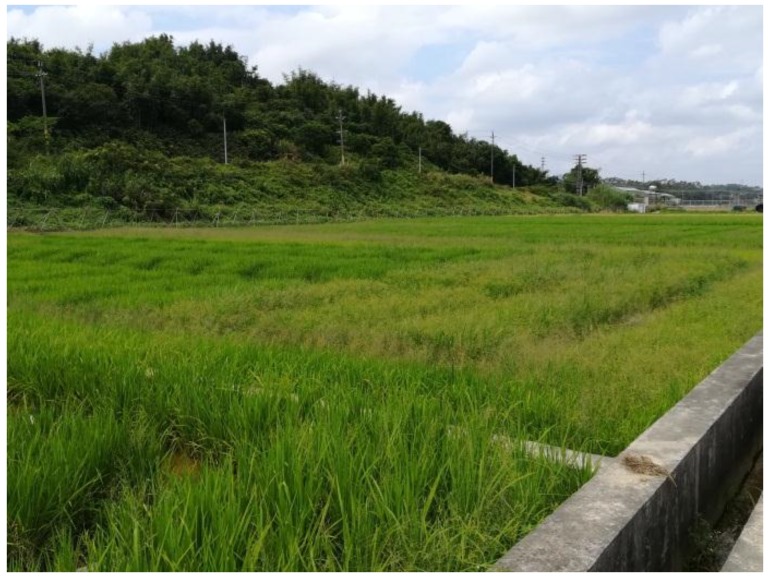
The photograph of the studied rice field.

Experiments were conducted in a rice field located in Guangdong Province, China (23°14′25″ N, 113°38′12″ E, in reference system datum WGS84). The field was a rectangle area of 90 × 60 m with flat ground. The seeds were sown on 21 August 2017, and the rice started to emerge 15 days after sowing. The study plot was naturally infested with *Cyperus iric* [23] and *L. chinensis* [24]. The photograph of the studied field is illustrated in Figure 1. 

Data collection was carried out on 2nd and 10th October 2017, when the rice and weeds were both in their early growth stages, and when the herbicide treatment was recommended. Phantom 4 (SZ DJI Technology Co., Ltd., Shenzhen, China) was used for data collection, and a 50 × 60 m plot was delimited in order to perform the flights. During the experiments, the flight altitude was set to 10 m, with a resolution of 0.5 cm per pixel. Sequences with a 70% forward-lap and 60% side-lap imagery were collected to cover the entire experimental plot. On 2nd and 10th October, 54 and 50 imagery (3000 × 4000 pixels) were collected in the experiments, respectively.

### 2.2. Dataset Preparation

Image mosaicking is an important step prior to image analysis [4]. In this work, the collected imagery were stitched together to form the ortho-mosaicked imagery using the software of Photoscan [25]. However, the ortho-mosaicked imagery is usually quite large (14,000 × 13,000 pixels in our work), making it a difficult task to carry out the data processing with limited CPU and GPU memory. In order to address this problem and to retain the original spatial resolution, we split the ortho-mosaicked imagery into small patches (1000 × 1000 pixels), similar with the work of Zhang et al. [26]. Following this strategy, the datasets of D02-1 and D10-1 were generated from the ortho-mosaicked imagery obtained on 2nd and 10th October 2017. Besides that, we also directly split the collected imagery into small patches (1000 × 1000 pixels), which generated the dataset of D02-2 and D10-2, as shown in Table 1.

**Figure 2:**
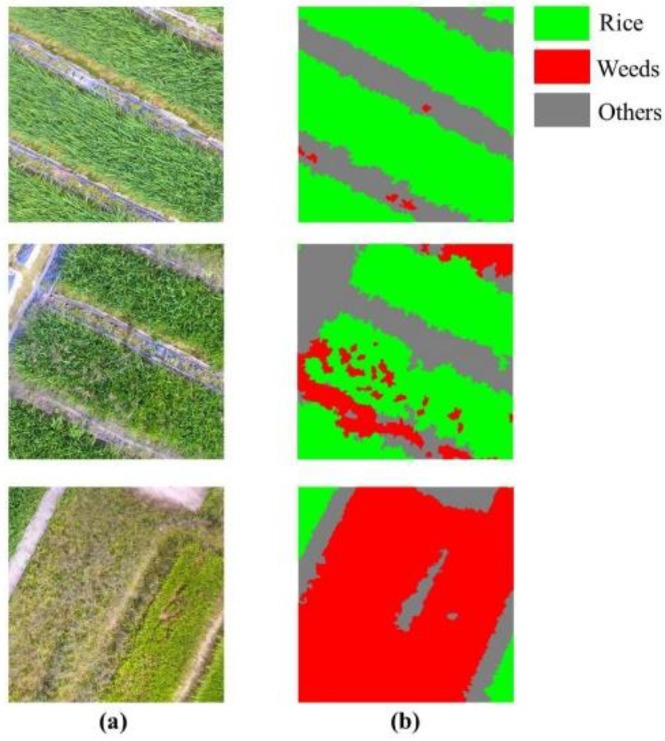
Three image-ground truth (GT) label pairs in the dataset: (**a**) images in the dataset; (**b**) corresponding GT labels.

For each imagery in the dataset, its corresponding ground truth (GT) label data was produced by careful manual labeling. With the high spatial resolution of UAV imagery, the weed-crop discrimination can be visually accessed, making it feasible to manually label the imagery at a pixel level. Thus, each sample in the dataset represented one image-GT label pair, and the GT label is used as the standard when evaluating the performance of the classifiers. Three image-GT label pairs are illustrated in Figure 2.

## 3. Methodology

In this work, two different workflows were applied to produce the weed cover map for the whole field. The performance of both workflows were evaluated and compared. Fully convolutional networks (FCN) was employed for the pixel level classification. Finally, the chessboard segmentation method was used to produce the prescription map based on the weed cover map.

### 3.1. Workflow

**Figure 3:**
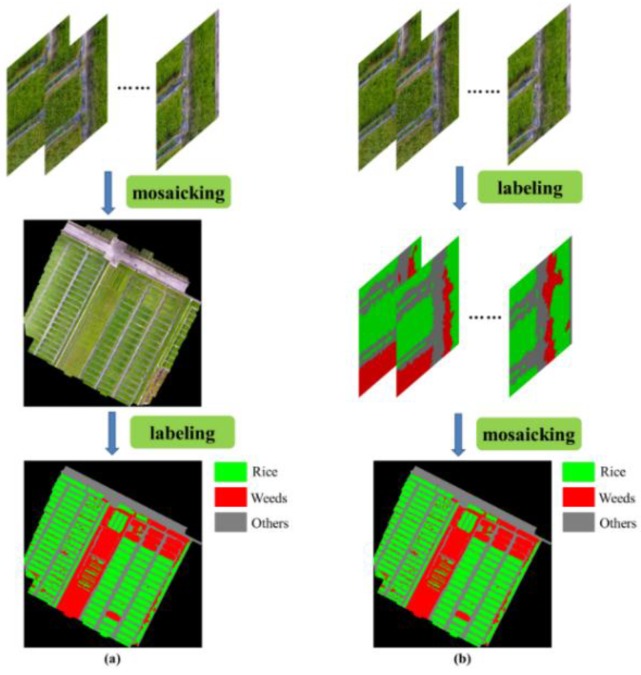
Two workflows to produce the weed cover map for the whole field. (**a**) The workflow of mosaicking-labeling; (**b**) the workflow of labeling-mosaicking.

Two different workflows were adopted as candidates, as shown in Figure 3. The first workflow conducted the mosaicking operation to generate the ortho-mosaicked imagery for the whole field, and then it performed a per-pixel classification to create the weed cover map. Inspired by the fact that some sections in the ortho-mosaicked imagery were blurring, which made it difficult to distinguish and may cause misclassification during the classification stage, we directly applied the labeling process on the collected imagery in the second workflow, which may avoid the ambiguous pixels in the classification stage. After that, the mosaicking process was conducted on the classification results, using the geo-information in the collected imagery. All of the mosaicking operations were performed using the software of Photoscan. 

The evaluation of the workflows was measured for accuracy and efficiency. The accuracy was evaluated by the overall accuracy and the mean intersection over union (mean IU) [16], and the time efficiency was measured using the total time required to generate the weed cover map, including data collection and data processing.

### 3.2. Semantic Labeling

Classical FCN-8s was proven to be effective on weed mapping of UAV imagery [21], which outperformed the traditional methods in terms of accuracy and efficiency. In this work, we sought for an optimal network architecture that will bring about a performance improvement. 

**Figure 4:**
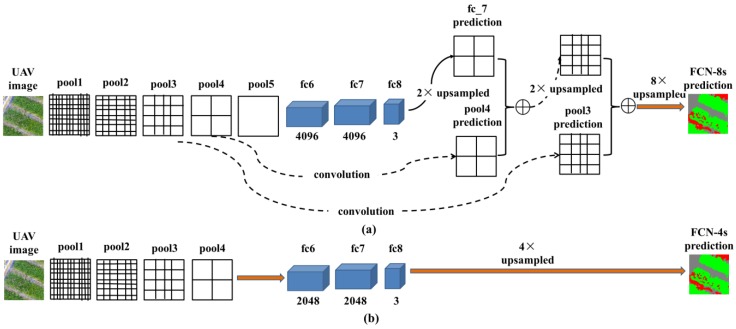
The illustration of the architecture of fully convolutional networks (FCN): (**a**) architecture of classical FCN-8s; (**b**) architecture of the modified FCN-4s.

Similar with the network architecture of classical FCN-8s [16], an ImageNet pre-trained Convolutional Neural Network (CNN) [27] was adapted to fully convolutional networks and was transferred to our study using a fine-tuning technique. Besides that, two modifications were conducted on the baseline architecture of FCN-8s. (1) In the previous experiments on the skip architecture [21], it was proven that the fusion of the prediction results (fc8) and the shallow layer of pool4 can effectively increase the prediction accuracy, as shown in Figure 4a. However, the fusion with other shallow layers brings no performance boost. This result indicated that the information from pool4 is crucial for the classification task, so that the fusion with this layer can make up the information loss caused by the downsampling operation. However, this strategy cannot properly address this problem, which resulted in low precision and blurred edges in the classification result [26]. Based on this result, the skip architecture and the last pooling operation (pool5) were removed so as to avoid the information loss of the layer of pool4. (2) The original network was designed for the dataset of PASCAL VOC 2011 segmentation challenge [28], which has 1000 different classes. However, our dataset only has three categories (rice, weeds, and others). According to the work of Stathakis et al. [29], there should be a positive correlation between the number of output classes and the number of neurons in the intermediate fully connected layers. Based on this theory, the number of feature maps of intermediate layers (fc6 and fc7, which were transformed from the fully connected layers) was reduced. The number of feature maps of the intermediate layers (fc6 and fc7) was set to 2048 through several experiments and an evaluation on the validation set (refer to Section 4). The network architecture of classical FCN-8s and the modified FCN-4s can be seen from Figure 4. 

Besides the modified FCN-4s, the classical FCN-8s [21] and Deeplab [22] were also applied and evaluated as comparison. For the FCN-8s, an ImageNet pre-trained CNN [27] was applied as a baseline architecture. The final classification layer was removed, and all of the fully connected layers were converted to convolutions. Skip architecture was built to improve the prediction precision. The lower layers (pool4 and pool5) were fused with the higher layer (fc8), as shown in Figure 4a. For the Deeplab approach, a 101-layer ResNet [30] was adapted in fully convolutional forms, similar with the approach of FCN-8s. The weights pre-trained on ImageNet [30] were transferred to our dataset using fine-tuning. Atrous convolution [17] was applied to extend the field of view (FOW) of the convolutional filters, and the fully connected random filed (CRF) [31] was used to further improve the prediction accuracy.

In this section, the accuracy was evaluated by the overall accuracy and mean intersection over union (mean IU) [16], similar to Section 3.1. However, the time efficiency was also measured using the processing time for one single image, which is the normal way for the evaluation adopted by most semantic labeling approaches [16,17].

### 3.3. Prescription Map Generation

The prescription map can be generated from the weed cover map. According to the work of López-Granados [4], a chessboard segmentation process was applied to build a grid framework of the prescription map. The weed cover map was split into small grids, and the comparison between the weed coverage of each grid and a given threshold was conducted: if the weed coverage of the grid is larger than the threshold value, it will be marked as a treatment area, otherwise it will be marked as a non-treatment area. The grid size is adjustable according to the different spraying machines, and it was set to 0.5 × 0.5 m in this work, in accordance with the site-specific sprayer [32].

For this section, the accuracy was calculated from two prescription maps (one generated from the weed cover map output by our algorithm, and the other from the GT label), which can be given by the following:

(1) $$\;\mathrm{accuracy}=\;\frac{The\;number\;of\;grid-based\;areas\;correcly\;classified}{The\;number\;of\;all\;grid-based\;areas}\;$$

For each prescription map, its relevant herbicide saving was calculated. According to the work of de Castro [8], herbicide saving is calculated in terms of the non-treatment area, which can be given by the following:

(2) $$\;\mathrm{herbicide}\;\mathrm{saving}=P_{non}\;=1-P_{treatment}\;$$

where $P_{non}$ and $P_{treatment}$ represent the proportion of the non-treatment and treatment areas.

## 4. Results and Discussions 

In this section, the experiments on workflows, semantic labeling, and prescription map generation will be conducted. In the experiments on workflows and semantic labeling approaches, the dataset was divided into training, validation, and testing set. The three datasets were used for parameter updating, hyper parameter tuning, and performance evaluation, respectively. All of the experiments were conducted on a computer with an Intel i7-7700 CPU and a NVIDIA GTX 1080 Ti GPU. During the process of weed mapping, the mosaicking operation was carried out in the CPU, while the semantic labeling approaches were performed using the GPU.

### 4.1. Workflow

In this section, two workflows (mosaicking-labeling and labeling-mosaicking) were applied in order to generate the weed cover map for the whole field. For the workflow of mosaicking-labeling, the dataset D02-1 (182 samples) was adopted as a training set. From dataset D10-1, 30% was randomly selected as validation set (54 samples), and the rest samples in the dataset D10-1 (128 samples) were used as the testing dataset. There were two reasons for this choice, namely: (1) the training set and testing set were chosen from different dates, which may evaluate the generalization capability of the algorithm, and (2) the validation set and testing set were selected from the same date, which may ensure that the two datasets belonged to the same distribution. For the workflow of labeling-mosaicking, the dataset D02-2 (648 samples) was used as training set, and 30% of dataset D10-2 (180 samples) was randomly selected as the validation set. However, we still used the testing set of the previous workflow (mosaicking-labeling) as the testing set of this workflow (labeling-mosaicking), since the classification on the ortho-mosaicked imagery is the ultimate objective of our algorithm.

In this section, the classical FCN-8s was used for the semantic labeling tasks. The quantitative results are listed in Table 2. From Table 2, it can be seen that both workflows obtained an approximate accuracy. However, because of the high overlapping in the collected imagery, directly processing on the collected imagery introduced too much redundant computation, which significantly lowered the inference speed. From this perspective, the workflow of mosaicking-labeling is the optimal solution, and will be considered as the default framework for the following experiments. 

### 4.2. Semantic Labeling

In this section, the dataset (training, validation and testing set) was the same as the workflow of mosaicking-labeling (Section 4.1). FCN-8s, Deeplab, and our modified FCN-4s were applied for our dataset, respectively. The quantitative results and confusion matrix by different approaches are shown in Table 3 and Table 4. From Table 3, it is obvious that Deeplab and FCN-4s outperformed FCN-8s in accuracy. From Table 4, it can be seen that the weed recognition rate of Deeplab and FCN-4s is above 0.90, which is higher than that of FCN-8s. There were two reasons possible for this result, namely: (1) the Deeplab used CRF to refine the spatial details, which increased the prediction accuracy, and (2) the FCN-4s removed the last pooling layer, which reduced the information loss and obtained performance boost. 

Although the Deeplab method achieved a satisfactory result for accuracy, the CRF introduced too much computation, which significantly slowed down the inference speed (Table 3). Therefore, it can be concluded that the FCN-4s strikes the best tradeoff between accuracy and efficiency. From Table 3, it can be found that the total time (including data collection and data processing) needed to generate the weed cover map for the entire field (50 × 60m) using FCN-4s is less than half an hour, demonstrating its rapid response capability on weed infestation monitoring.

**Figure 5:**
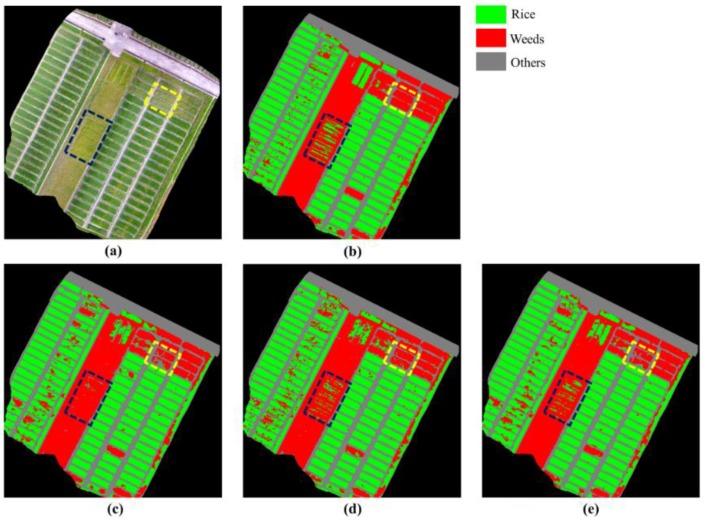
Weed cover maps output by different approaches. (**a**) Ortho-mosaicked imagery. (**b**) Corresponding GT-labels. The areas outside the studied plot were masked out (in black) and ignored in the training and evaluation. (**c**) Output by FCN-8s. (**d**) Output by Deeplab. (**e**) Output by FCN-4s.

The weed cover maps generated by the different approaches are shown in Figure 5. From Figure 5, it can be seen that (1) the weeds (in yellow dashed lines) were misclassified as others by FCN-8s, while they were properly recognized by Deeplab and FCN-4s; (2) the rice (in blue dashed lines) was misclassified as weeds by FCN-8s, while they were well classified by Deeplab and FCN-4s. From the qualitative results of Figure 5, it can be concluded that the FCN-4s obtained a satisfactory result with a simplified architecture in an end-to-end mode, which required no post-processing.

### 4.3. Prescription Map Generation

**Figure 6:**
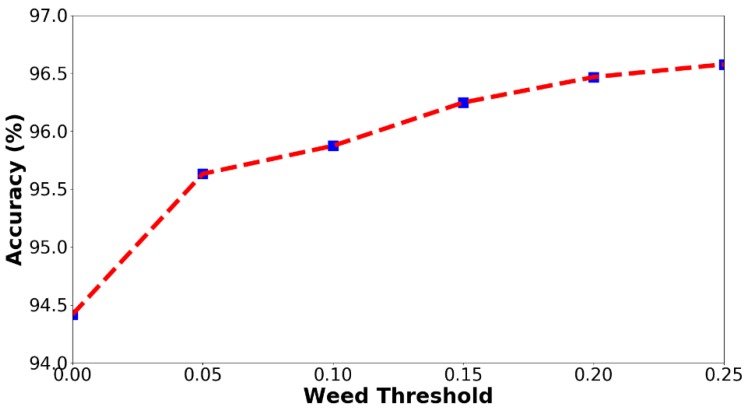
The accuracy curve with different weed thresholds.

The prescription map can be generated from a weed cover map with a given weed threshold. According to the experimental results in Table 3, the weed cover map obtained by FCN-4s was used to generate the prescription map. For a given weed threshold, the grid with a higher weed coverage will be marked as the treatment area. In this section, six thresholds (0.00–0.25, with an interval of 0.05) were evaluated. The accuracy using different weed thresholds is shown in Figure 6. From Figure 6, it can be seen that, with increasing threshold values, the accuracy consistently increases. The reason for this result is that large weed patches were easier for the classifiers to detect, thus resulting in a higher accuracy. High accuracies (above 0.94 for all thresholds) were observed from Figure 6, demonstrating that our algorithm is qualified for treatment area prediction. The treatment area and herbicide saving with different weed thresholds were calculated and are shown in Table 5. From Table 5, it can be seen that, with increasing the weed thresholds, the treatment area consistently decreases. The relevant herbicide saving ranges from 58.3% to 70.8%, demonstrating great potential to reduce the use of herbicides in SSWM applications. From a practical perspective, a threshold of 0.0 would be recommended as the optimal weed threshold for SSWM applications. There are two reasons for this choice, namely: (1) the accuracy (above 0.94) of this threshold is qualified and the relevant herbicide saving (58.3%) is acceptable, and (2) this threshold would minimize the risk of missing weed infestation, which may cause weed-crop competition.

**Figure 7:**
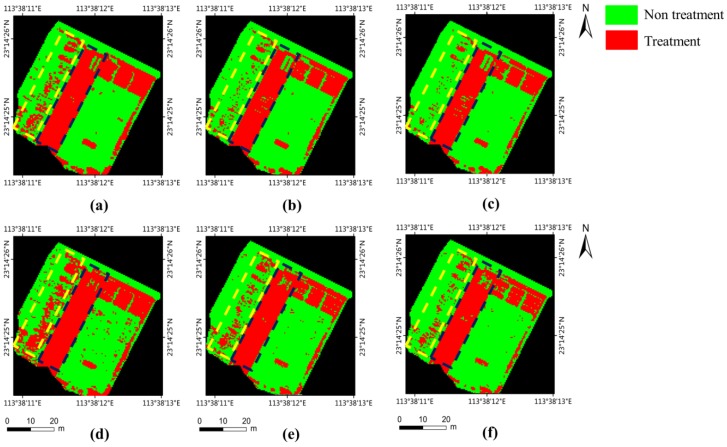
Prescription map generated with different weed thresholds. (**a**–**c**) Prescription map generated from the GT label using the weed thresholds of 0.0, 0.1 and 0.2. (**d**–**f**) Prescription map generated from the output weed cover map using the thresholds of 0.0, 0.1 and 0.2. From reference system datum WGS84.

The prescription maps generated with different thresholds are illustrated in Figure 7. From Figure 7, it can be seen that the changes of the threshold value have little influence on the areas with a high weed coverage (in blue dashed lines), as the weed coverage of these areas is higher than all of the threshold values. However, for the areas with a lower weed coverage (in yellow dashed lines), the weed threshold can effectively adjust the treatment areas, as the areas with lower weed coverage than the threshold will be ignored. The prescription maps generated by our method (with all thresholds) generally correspond to that generated by the GT label, thanks to the high accuracy of the output weed cover map. From Figure 7, it can also be seen that an overestimation for treatment areas was observed in the results of all of the thresholds. However, from an agronomic perspective, it is acceptable, as it can reduce the risk allowing the weeds to go untreated [33].

## 5. Conclusions

Prescription maps can provide decision making support for the spraying machine, which may effectively reduce the use of herbicide while enhancing the chemical effects. In this paper, the study on weed mapping and prescription map generation was conducted using UAV imagery. (1) The UAV imagery over a rice field were captured at a high spatial resolution, and pre-processing was performed so as to generate our dataset. (2) Two different workflows (mosaicking-labeling and labeling-mosaicking) were applied in order to generate the weed cover maps. These workflows were evaluated and compared. The experimental results showed that the workflow of mosaicking-labeling outperformed the others in terms of efficiency with an approximate accuracy. (3) A modified FCN-4s introduced pixel-to-pixel translation from UAV imagery to weed cover maps. Theoretic analysis was conducted to seek for architecture improvement. The improved architecture was evaluated and compared with the classical FCN-8s and Deeplab. The experimental results showed that the modified FCN-4s outperformed others in both accuracy and efficiency. (4) A chessboard segmentation method was used to build the grid framework of the prescription map. Different weed thresholds were applied and evaluated. High accuracies (above 0.94) were observed for all of the thresholds, and the relevant herbicide savings ranged from 58.3% to 70.8%. The method applied in this paper was superior in efficiency, which may produce a prescription map for a rice field (50 × 60 m) within half an hour, demonstrating its rapid response capability to the emergency of weed infestation. 

However, for the study of weed mapping and prescription map generation, more data is needed to extend and evaluate the generalization capability of the algorithm. Besides rough weed recognition, classification for specific weed species is also important for the SSWM applications, which can be extended based on our current work. All of these issues will be left as our future work.

## Figures and Tables

**Table 1:** Specification for the dataset.

Name	Flight Date	Number of Patches	Description
D02-1	2nd October 2017	182	Divided from the ortho-mosaic imagery
D10-1	10th October 2017	182	Divided from the ortho-mosaic imagery
D02-2	2nd October 2017	648	Divided from the collected imagery
D10-2	10th October 2017	600	Divided from the collected imagery

**Table 2:** Experimental results of different workflows. The speed was measured using the total time required to generate the weed cover map for the whole field, including data collection and data processing. Mean IU-mean intersection over union.

Workflow	Overall Accuracy	Mean IU	Speed
Mosaicking–labeling	0.9096	0.8303	24.8 min
Labeling–mosaicking	0.9074	0.8264	32.5 min

**Table 3:** Experimental results on different semantic labeling approaches. Speed-1 was measured using the inference time for a single imagery (1000 × 1000 pixels), and speed-2 was measured using the total time required to generate the weed cover map for the whole field, including data collection and data processing. FCN—fully convolutional networks.

Method	Overall Accuracy	Mean IU	Speed-1	Speed-2
FCN-8s	0.9096	0.8303	0.413 s	24.8 min
Deeplab	0.9191	0.8460	5.279 s	39.6 min
FCN-4s	0.9196	0.8473	0.356 s	24.7 min

**Table 4:** Confusion matrix by different semantic labeling approaches. GT—ground truth.

Method	GT/Predicted Category	Others	Rice	Weeds
FCN-8s	others	**0.939**	0.042	0.018
	rice	0.037	**0.894**	0.069
	weeds	0.078	0.027	**0.895**
Deeplab	others	**0.922**	0.044	0.034
	rice	0.023	**0.924**	0.052
	weeds	0.056	0.036	**0.907**
FCN-4s	others	**0.938**	0.030	0.031
	rice	0.037	**0.913**	0.049
	weeds	0.055	0.039	**0.905**

**Table 5:** Herbicide saving with different weed thresholds.

Threshold	Treatment Area	Herbicide Saving
0.00	41.7%	58.3%
0.05	35.9%	64.1%
0.10	33.6%	66.4%
0.15	31.9%	68.1%
0.20	30.4%	69.6%
0.25	29.2%	70.8%
